# Dynamic changes of D-dimer and neutrophil-lymphocyte count ratio as prognostic biomarkers in COVID-19

**DOI:** 10.1186/s12931-020-01428-7

**Published:** 2020-07-03

**Authors:** Wenjing Ye, Guoxi Chen, Xiaopan Li, Xing Lan, Chen Ji, Min Hou, Di Zhang, Guangwang Zeng, Yaling Wang, Cheng Xu, Weiwei Lu, Ruolin Cui, Yuyang Cai, Hai Huang, Ling Yang

**Affiliations:** 1 Department of Respiratory Medicine, Xinhua Hospital, School of Medicine, Shanghai Jiao Tong University, Shanghai, 200092 China; 2 Department of Tuberculosis ward 2, Wuhan Pulmonary Hospital, Wuhan, 430030 Hubei China; 3 Center for Disease Control and Prevention, Pudong New Area, Shanghai, 200136 China; 4 Fudan University Pudong Institute of Preventive Medicine, Pudong New Area, Shanghai, 200136 China; 5 Warwick Clinical Trials Unit, Warwick Medical School, Coventry, UK; 6 School of Public Health, Shanghai Jiao Tong University School of Medicine, Shanghai, 200025 China; 7 Department of Geriatrics, Xinhua Hospital, School of Medicine, Institute of Hospital Development Strategy, China Hospital Development Institute, Shanghai Jiao Tong University, Shanghai, 200092 China; 8 Department of Emergency, Xinhua Hospital, School of Medicine, Shanghai Jiao Tong University, Shanghai, 200092 China; 9 Department of Chinese Medicine, Xinhua Hospital, School of Medicine, Shanghai Jiao Tong University, Shanghai, 200092 China

**Keywords:** COVID-19, D-dimer, Neutrophil-lymphocyte count ratio, Prognostic biomarker, Cox regression

## Abstract

**Background:**

Since December 2019, the outbreak of COVID-19 caused a large number of hospital admissions in China. Many patients with COVID-19 have symptoms of acute respiratory distress syndrome, even are in danger of death. This is the first study to evaluate dynamic changes of D-Dimer and Neutrophil-Lymphocyte Count Ratio (NLR) as a prognostic utility in patients with COVID-19 for clinical use.

**Methods:**

In a retrospective study, we collected data from 349 hospitalized patients who diagnosed as the infection of the COVID-19 in Wuhan Pulmonary Hospital. We used ROC curves and Cox regression analysis to explore critical value (optimal cut-off point associated with Youden index) and prognostic role of dynamic changes of D-Dimer and NLR.

**Results:**

Three hundred forty-nine participants were enrolled in this study and the mortality rate of the patients with laboratory diagnosed COVID-19 was 14.9%. The initial and peak value of D-Dimer and NLR in deceased patients were higher statistically compared with survivors (*P* < 0.001). There was a more significant upward trend of D-Dimer and NLR during hospitalization in the deceased patients, initial D-Dimer and NLR were lower than the peak tests (MD) -25.23, 95% CI: − 31.81- -18.64, *P* < 0.001; (MD) -43.73, 95% CI:-59.28- -31.17, *P* < 0.001. The test showed a stronger correlation between hospitalization days, PCT and peak D-Dimer than initial D-Dimer. The areas under the ROC curves of peak D-Dimer and peak NLR tests were higher than the initial tests (0.94(95%CI: 0.90–0.98) vs. 0.80 (95% CI: 0.73–0.87); 0.93 (95%CI:0.90–0.96) vs. 0.86 (95%CI:0.82–0.91). The critical value of initial D-Dimer, peak D-Dimer, initial NLR and peak NLR was 0.73 mg/L, 3.78 mg/L,7.13 and 14.31 respectively. 35 (10.03%) patients were intubated. In the intubated patients, initial and peak D-Dimer and NLR were much higher than non-intubated patients (*P* < 0.001). The critical value of initial D-Dimer, peak D-Dimer, initial NLR and peak NLR in prognosticate of intubation was 0.73 mg/L, 12.75 mg/L,7.28 and 27.55. The multivariable Cox regression analysis showed that age (HR 1.04, 95% CI 1.00–1.07, *P* = 0.01), the peak D-Dimer (HR 1.03, 95% CI 1.01–1.04, *P* < 0.001) were prognostic factors for COVID-19 patients’ death.

**Conclusions:**

To dynamically observe the ratio of D-Dimer and NLR was more valuable during the prognosis of COVID-19. The rising trend in D-Dimer and NLR, or the test results higher than the critical values may indicate a risk of death for participants with COVID-19.

## Background

A novel coronavirus (severe acute respiratory syndrome coronavirus 2, SARS-CoV-2) causing a cluster of respiratory infections (coronavirus disease 2019, COVID-19), was identified on 7 January 2020 in Wuhan, China [1–3]. In the following days, the 2019-nCoV has been quickly spreading in China and other countries. Up to March 23, 2020, a total of 285,179 cases had been confirmed, including 13,577 deaths, and the mortality rate was 4.76%. Because of its severity, it would be valuable to explore risk factors of the death in patients due to severe 2019-nCoV infected pneumonia at certain stages as earlier as possible, in order to timely take actions on, reasonable intervention to enhance the cure rate and the prognosis quality [4]. Neutrophil-Lymphocyte Ratio (NLR) is a convenient and quick index of inflammation detection in laboratory examination. It is used in the diagnosis, treatment and prognosis evaluation of pneumonia [5]. Several studies have showed that elevated plasma D-Dimer was a prognostic factor for adverse outcomes in respiratory diseases [6, 7]. The aim of our study was to evaluate the prognostic utility of dynamic changes of D-Dimer and NLR, especially the initial test on admission and peak value during hospitalization, in patients with COVID-19.

## Materials and methods

### Study design and Participants

We conducted a retrospective, single centred and observational study in Wuhan Pulmonary Hospital, Hubei Province, China (a COVID-19-designated hospital in the epidemic outbreak), and collected clinical data in patients with the diagnosed COVID-19 between January 1 and March 16, 2020. There were 408 patients in all. Fifty-nine patients who never took D-Dimer or routine blood test were excluded in this study. The diagnosis and treatment of COVID-19 complied with the “new coronary pneumonia diagnosis and treatment plan” issued by the health commission of the People’s Republic of China (reference). Laboratory diagnosis of COVID-19 are confirmed by viral nucleic acid test (NAT) using high-throughput sequencing or real-time reverse-transcriptase–polymerase-chain-reaction (RT-PCR) amplification of open reading frame 1ab (ORF1ab) and nucleocapsid protein (NP) genes fragments from sputum, pharyngeal swab or lower respiratory tract samples. Three hundred forty-nine patients were enrolled in this study, among whom 297 patients were survivors (survival group), while 52 died (death group).

The National Health Commission of the People’s Republic of China has determined that data collection and analysis of cases and close contacts are part of ongoing investigations into outbreaks of public health events and are therefore exempt from the approval requirements of the institutional review board.

### Data collection

Clinical data includes demographic information (gender, age, time of admission, time of discharge, comorbidities), laboratory tests (routine blood test, coagulation test, c-reaction protein (CRP), procalcitonin (PCT), troponin I (TNI), N-terminal pro-Brain Natriuretic Peptide (NT-proBNP)) and outcome (survival or death at hospital discharge). Coagulation function test includes fibrinogen (FIB), activated partial thromboplastin time (APTT), prothrombin time (PT) and D-Dimer. Multiple D-Dmier and blood routine tests were taken for each patient during hospitalization. We collected the complete results of these tests and extracted the initial and peak results on admission and post admission, respectively.

### Statistical analysis

SPSS version 24.0 (IBM Corp, Armonk, NY) was used for statistical analysis. Differences between qualitative variables were analyzed using Chi-squared test. Differences in the quantitative variables were analyzed using t-test or paired t-test. In the case of nonnormally distributed variables, the non-parametric Mann Whitney test was used. Pearson’s correlation coefficient was used to measure the correlation between variables. Receiver operating characteristic (ROC) curves were constructed to evaluate the sensitivity and specificity of initial D-Dimer, initial NLR, peak D-dimer, and peak NLR in predicting death. The area under the curve (AUC) was calculated, with higher values indicating better discriminatory ability. Subsequently, Cox regression model was used to explore the critical value and prognostic role of dynamic changes of D-Dimer and NLR. A *P* < 0.05 was considered statistically significant.

## Result

We included 349 participants in this study with a median age of 62 (interquartile range, (IQR): 21.00–69.00) years, and N (49.6%) were males. 35 (10.03%) patients were intubated. 297 (85.1%) patients survived to hospital discharge, while 52 (14.9%) died. The mean length of stay was 12 (IQR:7.32–17.00) days. Respiratory failure and acute respiratory distress syndrome (ARDS) were reported in 44 (12.6%) and 35 (10.0%) patients, respectively. Demographic characteristics, coagulation function test, infection markers (CRP, PCT), cardiac injury index (TNI, NT-proBNP) and comorbidities of all patients were shown in Table 1.

**Table 1:** Baseline characteristics and laboratory tests of 349 patients

		Total (*n* = 349)	Survivors (*n* = 297)	Death (*n* = 52)	*P*
Gender (Male,%)		173(49.60%)	137(46.10%)	36(69.2%)	0.002
Age (years), *n* = 349		62.00(21.00–69.00)	60.00(50.00–67.00)	69.00(63.25–75.50)	< 0.001
Hospitalization days (days), *n* = 349		12.00(7.32–17.00)	11.00(7.00–16.00)	14.00(9.00–23.75)	0.006
Initial D-Dimer (mg/L), *n* = 349		0.39(0.17–0.95)	0.35(0.15–0.62)	1.81(0.52–9.34)	< 0.001
FIB(g/L),*n* = 345		3.83(3.13–4.79)	3.83(3.22–4.82)	3.92(2.89–4.71)	0.349
APTT(s),*n* = 347		34.80(32.40–38.50)	34.90(32.40–38.20)	34.60(32.08–41.48)	0.488
PT(s),*n* = 347		13.00(12.40–13.90)	12.80(12.40–13.50)	14.30(12.93–15.80)	< 0.001
Initial NLR, *n* = 349		3.33(1.94–9.42)	2.88(1.79–6.74)	14.96(8.52–26.58)	< 0.001
CRP (mg/L),*n* = 276		18.59(0.99–61.84)	12.31(0.84–47.08)	65.45(25.81–98.90)	< 0.001
PCT (ng/ml), *n* = 250		0.04(0.04–0.05)	0.04(0.04–0.04)	0.12(0.04–0.39)	< 0.001
TNI (ng/ml),*n* = 215		0.03(0.01–0.03)	0.01(0.01–0.03)	0.03(0.03–0.12)	< 0.001
NT-proBNP (pg/ml),*n* = 240		102.00(23.00–442.25)	64.00(16.50–249.00)	709.00(322.00–1282.00)	< 0.001
Comorbidity(n,%)	Respiratory failure	44(12.60%)	17(5.70%)	27(51.90%)	< 0.001
	ARDS	35(10.00%)	11(3.70%)	24(46.20%)	< 0.001
	Heart failure	16(4.60%)	5(1.70%)	11(21.20%)	< 0.001
	AKI	14(4.00%)	1(0.30%)	13(25.00%)	< 0.001
	Hypertension	103(29.50%)	73(24.60%)	30(57.70%)	< 0.001
	Diabetes mellitus	57(16.30%)	41(13.80%)	16(30.80%)	0.004
	Hypoproteinemia	71(20.30%)	53(17.80%)	18(34.60%)	0.009

NOTE: Data are median (IQR), n (%). *FIB* fibrinogen; *APTT* activated partial thromboplastin time; *PT* prothrombin time; *NLR* neutrophil-to-lymphocyte ratio; *CRP* C-reactive protein; *PCT* procalcitonin; *TNI* troponin I; *NT*-*proBNP* N-terminal pro brain natriuretic peptide; *ARDS* acute respiratory distress syndrome; *AKI* acute kidney injury

### Dynamic changes of D-dimer, NLR and prognosis

In the deceased patients, initial D-Dimer and NLR were lower than the peak tests (MD) -25.23, 95% CI:-31.81- -18.64, *P* < 0.001; (MD) -43.73, 95% CI:-59.28- -31.17, *P* < 0.001. The initial and peak D-Dimer and NLR in the survivors were significantly lower compared with those of the deceased patients (Table 2). 35 (10.03%) patients were intubated. In the intubated patients, initial and peak D-Dimer and NLR were much higher than non-intubated patients, which had statistically significant differences (*P* < 0.001) (STable 1).

**Table 2:** Dynamic changes of D-Dimer, NLR and prognosis

	Survivors (*n* = 297)	Death (*n* = 52)	HR (95% CI)	*P* value
Initial D-Dimer (mg/L), *n* = 349	0.35(0.15–0.62)	1.81(0.52–9.34)	−6.61(−10.45- -2.77)	< 0.001
Peak D-Dimer (mg/L), *n* = 349	0.40(0.18–47.08)	29.44(12.23–61.77)	−31.10(−37.79- -24.41)	< 0.001
Initial NLR, *n* = 349	2.88(1.79–6.74)	14.96(8.52–26.58)	−13.55(−18.00- -9.10)	< 0.001
Peak NLR, *n* = 349	4.14(2.11–12.32)	46.58(27.95–87.29)	−52.83(−67.44- -38.23)	< 0.001

NOTE: Data are median (IQR). *NLR* neutrophil-to-lymphocyte ratio

### Dynamic changes of D-dimer, NLR and other laboratory tests

The associations between dynamic changes in D-Dimer, NLR and infection markers, cardiac injury index and hospitalization days were shown in Table 3. It could be found that there was a strong correlation between D-Dimer, NLR and other markers (*P* < 0.05). The test showed a stronger correlation between hospitalization days, PCT and peak D-Dimer than initial D-Dimer.

**Table 3:** Correlation study between DD, NLR and other laboratory tests

	Age (years)	CRP (mg/L)	PCT (ng/ml)	TNI (ng/ml)	NT-proBNP (pg/ml)	Hospitalization days (days)
Initial D-Dimer,mg/L, (PCC,*P*)	0.20,*P* < 0.001	0.14,*P* = 0.007	0.09,*P* = 0.1	0.61,*P* < 0.001	0.26,*P* < 0.001	0.04,*P* = 0.5
Peak D-Dimer, mg/L, (PCC,*P*)	0.23,*P* < 0.001	0.36,*P* < 0.001	0.25,*P* < 0.001	0.27,*P* < 0.001	0.34,*P* < 0.001	0.21,*P* < 0.001
Initial NLR (PCC,*P*)	0.28,*P* < 0.001	0.55,*P* < 0.001	0.58,*P* < 0.001	0.14,*P* = 0.04	0.44,*P* < 0.001	0.24,*P* < 0.001
Peak NLR (PCC,*P*)	0.29,*P* < 0.001	0.42,*P* < 0.001	0.30,*P* < 0.001	0.13,*P* = 0.04	0.41,*P* < 0.001	0.31,*P* < 0.001

NOTE: *PCC* Pearson Correlation Coefficient; *CRP* c-reaction protein; *PCT* procalcitonin; *TNI* troponin I; *NT-proBNP* N-terminal pro-Brain Natriuretic Peptide; *NLR* neutrophil-lymphocyte ratio

### Predictive accuracy of dynamic changes of D-dimer, NLR

**Fig. 1:**
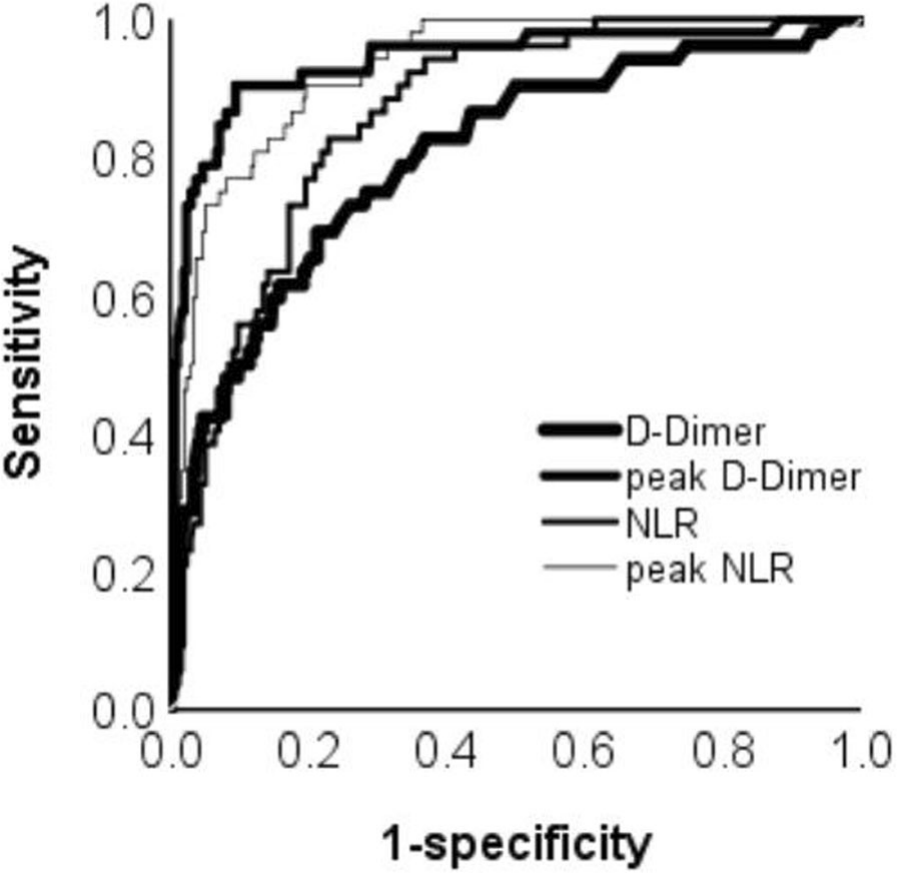
ROC curves of D-dimer and NRL as an overall predictor of death

Figure 1 showed that the area under the curve of initial D-Dimer, peak D-Dimer, initial NLR and peak NLR were more than 0.7, so they are good the predictive values. The peak D-Dimer and peak NLR tests were higher than the initial tests (0.94(95%CI: 0.90–0.98) vs. 0.80 (95% CI: 0.73–0.87); 0.93 (95%CI:0.90–0.96) vs. 0.86 (95%CI:0.82–0.91). The critical value of initial D-Dimer, peak D-Dimer, initial NLR and peak NLR was 0.73 mg/L, 3.78 mg/L,7.13 and 14.31 (Table 4). Using the same statistical method, we found that the critical value of initial D-Dimer, peak D-Dimer, initial NLR and peak NLR in prognosticate of intubation was 0.73 mg/L, 12.75 mg/L,7.28 and 27.55.

**Table 4:** Area under the curve and critical value of D-Dimer and NLR

Parameter	AUC (95% CI)	critical value
Initial D-Dimer	0.80 (0.73–0.87)	0.73
Peak D-Dimer	0.94 (0.90–0.98)	3.78
Initial NLR	0.86 (0.82–0.91)	7.13
Peak NLR	0.93 (0.90–0.96)	14.31

NOTE:*AUC* area under the curve; *NLR* neutrophil-to-lymphocyte ratio

### Cox regression analysis of patient outcomes

**Fig. 2:**
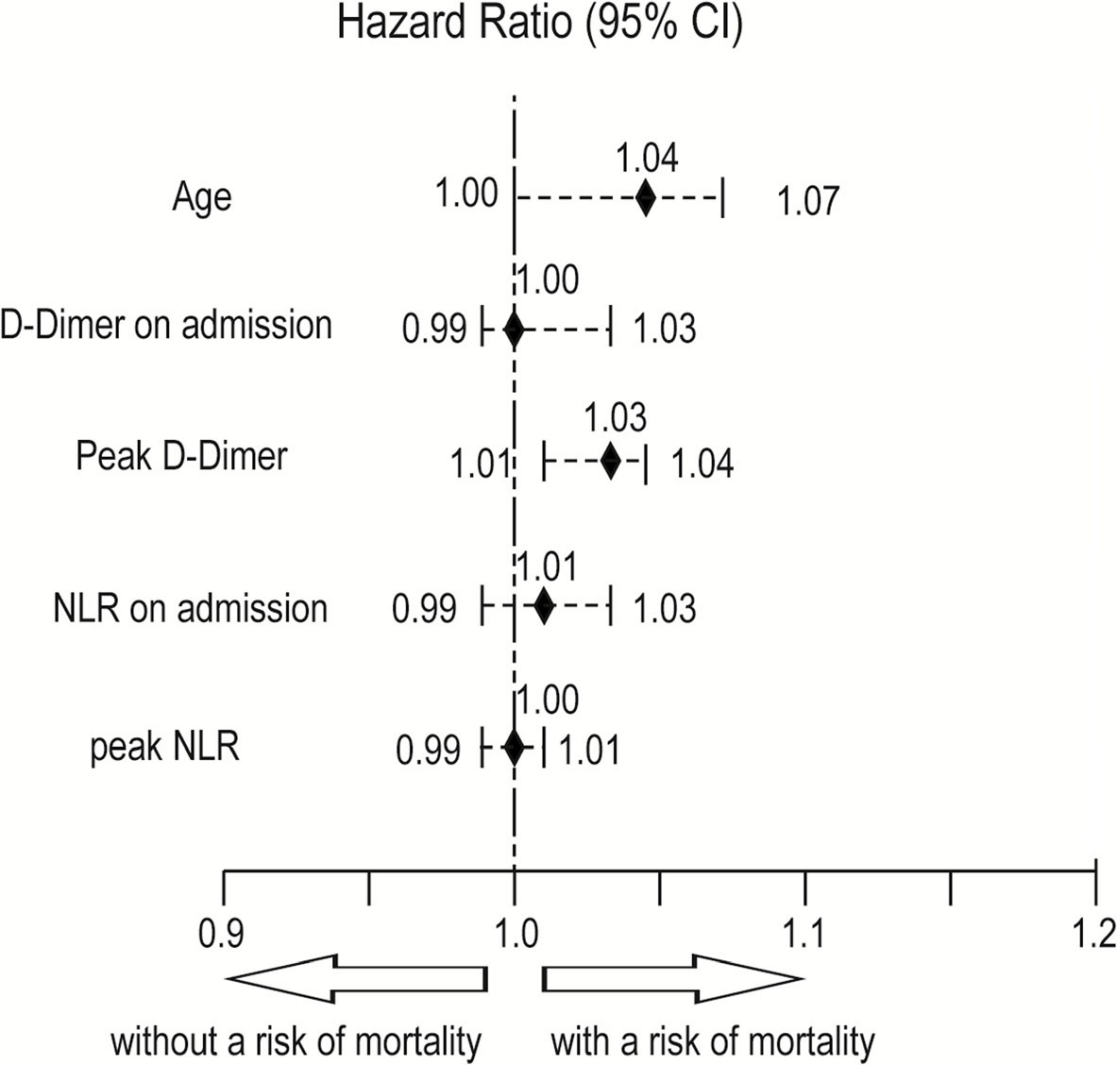
Multivariate Cox regression analysis of potential prognostic factors

The Cox regression analysis showed that age (HR 1.04, 95% CI 1.00–1.07, *P* = 0.01), the peak D-Dimer (HR 1.03, 95% CI 1.01–1.04, *P* < 0.001) were prognostic factors for COVID-19 patients’ death (Fig. 2). Higher peak D-Dimer and elder ages were associated with a higher risk of mortality.

## Discussion

Since December 2019, some cases with unknown pneumonia were reported in Wuhan with exposure to a large Hua’nan seafood market. It has been identified as an acute respiratory infection caused by a novel coronavirus, later named as Covid-19 by the World Health Organization. So far, confirmed cases have been found in many countries or regions [8]. Covid-19 causes severe illness and sustained person-to-person transmission, making it a concerning and serious public health threat. It is important for health professionals to be aware of this new 2019-nCoV so that coordinated, timely, and effective actions can be taken to help prevent additional cases or poor health outcomes [9].

D-Dimer is a specific degradation product that is produced in hydrolysis of fibrin [10].. It may reflect the effects of infection on coagulation in infectious diseases. Some studies reported increase in D-Dimer levels in patients with pneumonia, has an indication of the blood hypercoagulable state and the presence of thrombosis [11, 12]. D-Dimer of critically ill patients with COVID-19 was significantly increased, with frequent clotting disorders and microthrombotic formation in peripheral blood vessels [8]. In this retrospective study, we not only assessed the single D-Dimer value, but also investigated the dynamic changes of D-Dimer, especially the significance of peak value for prognosis.

We found that in the deceased patients, peak D-Dimer and NLR were higher than the initial tests. The dynamic changes of D-Dimer and NLR in survival group were statistically significant compared with deceased patients, and the value in deceased patients was higher than those in survival group. Additionally, Peak D-Dimer value was significantly correlated with hospitalization days and PCT rather than initial D-Dimer. We also identified the optimal cut-off points of initial and peak D-Dimer for defining the patients with high risk of death. If the initial D-Dimer of patients was higher than 0.73 mg/L, or the peak D-Dimer increased was higher than 3.78 mg/L, patients could be considered of high risk of death and require further intensive and immediate treatments to prevent a devastating outcome. By using the Cox regression analysis, we found that the peak value of D-Dimer, rather than the initial D-Dimer, was the risk factor of the prognosis of patients with COVID-19. Patients with higher peak D-Dimer value tended to have a higher risk of death. High D-Dimer is likely to be associated with persistent clotting disorders, microthrombotic formation, pulmonary embolism and acute myocardial infarction in long-stay or death patients, which may cause refractory hypoxemia, respiratory failure, disseminated intravascular coagulation or death.

Neutrophil-lymphocyte ratio is a marker of systemic inflammation that has a quick and simple operation, and predicts prognosis in various pathological conditions [13, 14]. Recently, NLR was found to have greater prognostic power than traditional infection markers, such as CRP, white blood cell count and neutrophil count, in community-acquired pneumonia [15, 16]. At the early stage of COVID-19, the total number of leukocytes in peripheral blood is normal or decreases, while the lymphocyte count decreases [8]. However, it is not clear how lymphocyte count changes as the disease progresses. We analyzed the dynamic changes of NLR in this study. It was found that both the initial and the peak values had a good correlation with the infection markers (CRP, PCT). Therefore, it could be used as the basis to assess the severity of infection. The initial NLR had a lower AUC compared to the peak NLR, suggesting the peak value is a better predictive marker for death outcome. The critical values of initial NRL and peak NRL were 7.13 and 14.31 respectively. The increase of NLR means the progressive increase of neutrophils, and/or the decrease of lymphocyte. The increase of neutrophils often indicates that the patients have bacterial infection and the infection is aggravated. The decrease of lymphocyte means that the immune function is poor [13, 14]. Those suggest that the aggravated condition and the infection is difficult to control. Generally, it is necessary to pay attention to the COVID-19 patients with increased NLR, who may have a poor prognosis, even a risk of death.

The inflammatory response is thought to underpin COVID-19 pathogenesis. The sudden clinical deterioration 1 week after initial symptom onset suggests that severe respiratory failure in COVID-19 is driven by a unique pattern of immune dysfunction. Unique pattern of immune dysregulation in severe COVID-19 is associated with sustained cytokine production and hyper-inflammation [17, 18]. These studies remind us that in addition to DD and NLR, we also need to pay more attention to the dynamic changes of cytokines and immune function in later studies, observe the time of peak occurrence and the correlation with the severity of disease.

This study had some limitations. First, this is a single center retrospective study. All of data were collected from patients in Wuhan Pulmonary Hospital. Most of patients in this hospital were symptomatic, severe or even critical, the mortality rate of which reached 14.9%. Asymptomatic and mild patients could have been missed. So the results may not be representative for all types, especially asymptomatic and mild types. Second, the peak values detected in the laboratory might not be the true peak values in the course of disease. Some patients were critically ill and died rapidly after admission. They may have only one blood sampling result, and we were unable to observe their changes of D-Dimer and NLR. In addition, some patients might have been in recovery when being admitted to hospital. Therefore, we might have missed the chance to take the blood test at their most serious stage of the disease. Thus, the results might not fully reflect the disease progression. However, our results showed much higher values of the initial D-Dimer and NRL in the deceased patients than in the survival group. These values were related to CRP, PCT, TnI and hospitalization days. Under certain conditions, high D-Dimer and high NRL can effectively indicate the poor prognosis. In addition, we studied the critical values. If the test result of patients was higher than the critical value, it might indicate that the prognosis of patients was poor. Third, this study was limited due to medical resources. For example, Pulmonary embolism is usually to be investigated when D-Dimer is elevated. However, these markers were not available in the medical records. Recently, most patients only completed regular chest computed tomography, rather than computed tomographic pulmonary angiography (CTPA). Therefore, It was difficult to further analyze the cause of D-Dimer elevation. Further studies are needed to analyze the cause of D-Dimer and NLR changes in order to improve the understanding of the infection and provide effective treatment to reduce the COVID-19 mortality. Last, our data may be subjected to recall bias and selection bias due to the nature of our study. For example, the record of patients’ comorbidities might not be accurate and complete, considering the unprecedented pressure during admission and treatment. Further studies with more detailed and representative data are needed.

## Conclusions

Multi-tests of D-Dimer and NLR were more valuable than single tests to treat patients with the infection of COVID-19, due to higher values of D-Dimer and NLR in deceased patients than survival group. Further studies are needed to analyze the cause of D-Dimer and NLR changes in order to provide potential treatment for the COVID-19 infection and decrease mortality.

## Supplementary information

**Additional file 1: Table S1.** Dynamic changes of D-Dimer, NLR in intubated and non-intubated patients

## Data Availability

The datasets used during the current study are available from the corresponding author on reasonable request.

## Abbreviations

NLR: Neutrophil-lymphocyte count ratio
SARS-CoV-2: Severe acute respiratory syndrome coronavirus 2
COVID-19: Coronavirus disease 2019
NAT: Nucleic acid test
RT-PCR: Real-time reverse-transcriptase–polymerase-chain-reaction
CRP: C-reaction protein
PCT: Procalcitonin
TNI: Troponin I
NT-proBNP: N-terminal pro-Brain Natriuretic Peptide
FIB: Fibrinogen
APTT: Activated partial thromboplastin time
PT: Prothrombin time
ROC: Receiver operating characteristic
AUC: Area under the curve
IQR: Interquartile range
CTPA: Computed tomographic pulmonary angiography
