# Supplementary material for: Dynamic changes of D-dimer and neutrophil-lymphocyte count ratio as prognostic biomarkers in COVID-19
Source: Respir Res. 2020 Jul 3;21:169. doi: 10.1186/s12931-020-01428-7 (PMC7332531; doi:10.1186/s12931-020-01428-7)
Supplement: Supplementary file 1 — Additional file 1: Table S1. Dynamic changes of D-Dimer, NLR in intubated and non-intubated patients [file 12931_2020_1428_MOESM1_ESM.docx]

STable 1. Dynamic changes of D-Dimer, NLR in intubated and non-intubated patients

|  | **Non-intubated patient (n=314)** | **Intubated patient (n=35)** | ***P* value** |
| --- | --- | --- | --- |
| **Initial D-Dimer (mg/L)** | 0.35(0.16-0.66) | 1.98(0.59-13.90) | <0.001 |
| **Peak D-Dimer(mg/L),** | 0.42(0.18-1.34) | 32.79(20.78-64.01) | <0.001 |
| **Initial NLR** | 3.01(1.84-7.36) | 16.06(8.54-26.83) | <0.001 |
| **Peak NLR** | 4.75(2.16-13.52) | 52.75(38.33-87.39) | <0.001 |

NOTE: Data are median (IQR). NLR,neutrophil-to-lymphocyte ratio
